# Investigating Effects of Tulathromycin Metaphylaxis on the Fecal Resistome and Microbiome of Commercial Feedlot Cattle Early in the Feeding Period

**DOI:** 10.3389/fmicb.2018.01715

**Published:** 2018-07-30

**Authors:** Enrique Doster, Pablo Rovira, Noelle R. Noyes, Brandy A. Burgess, Xiang Yang, Margaret D. Weinroth, Steven M. Lakin, Christopher J. Dean, Lyndsey Linke, Roberta Magnuson, Kenneth I. Jones, Christina Boucher, Jamie Ruiz, Keith E. Belk, Paul S. Morley

**Affiliations:** ^1^Microbial Ecology Group, Colorado State University, Fort Collins, CO, United States; ^2^Department of Microbiology, Immunology and Pathology, Colorado State University, Fort Collins, CO, United States; ^3^Department of Animal Sciences, Colorado State University, Fort Collins, CO, United States; ^4^Department of Veterinary Population Medicine, University of Minnesota, St. Paul, MI, United States; ^5^Department of Population Health, University of Georgia, Athens, GA, United States; ^6^Department of Clinical Sciences, Colorado State University, Fort Collins, CO, United States; ^7^Department of Biochemistry and Molecular Genetics, University of Colorado Denver School of Medicine, Aurora, CO, United States; ^8^Department of Computer and Information Science and Engineering, University of Florida, Gainesville, FL, United States

**Keywords:** feedlot, metaphylaxis, tulathromycin, metagenomics, microbiome, resistome

## Abstract

The objective was to examine effects of treating commercial beef feedlot cattle with therapeutic doses of tulathromycin, a macrolide antimicrobial drug, on changes in the fecal resistome and microbiome using shotgun metagenomic sequencing. Two pens of cattle were used, with all cattle in one pen receiving metaphylaxis treatment (800 mg subcutaneous tulathromycin) at arrival to the feedlot, and all cattle in the other pen remaining unexposed to parenteral antibiotics throughout the study period. Fecal samples were collected from 15 selected cattle in each group just prior to treatment (Day 1), and again 11 days later (Day 11). Shotgun sequencing was performed on isolated metagenomic DNA, and reads were aligned to a resistance and a taxonomic database to identify alignments to antimicrobial resistance (AMR) gene accessions and microbiome content. Overall, we identified AMR genes accessions encompassing 9 classes of AMR drugs and encoding 24 unique AMR mechanisms. Statistical analysis was used to identify differences in the resistome and microbiome between the untreated and treated groups at both timepoints, as well as over time. Based on composition and ordination analyses, the resistome and microbiome were not significantly different between the two groups on Day 1 or on Day 11. However, both the resistome and microbiome changed significantly between these two sampling dates. These results indicate that the transition into the feedlot—and associated changes in diet, geography, conspecific exposure, and environment—may exert a greater influence over the fecal resistome and microbiome of feedlot cattle than common metaphylactic antimicrobial drug treatment.

## Introduction

One of the most critical periods in managing the health and wellbeing of beef cattle is when they are transitioned from less intensive production settings, such as pasture or backgrounding operations, to feedlots. During this transition, animals are exposed to varied stressors associated with handling, transport, processing, commingling, and a shift to a high-energy feedlot diet (Sanderson et al., 2008). In response to these stressors, animals may become more susceptible to infectious disease, such as those that cause bovine respiratory disease (BRD), the single largest cause of morbidity and mortality among feedlot cattle in the United States (Hilton, 2014; Grissett et al., 2015). Because groups of cattle that are deemed to have an especially high risk of BRD can already be incubating infections that will become life-threatening despite the absence of clinical signs, groups of cattle with particularly high risk of BRD are sometimes treated with antimicrobial drugs (AMDs) at the time they enter feedlots, a practice that is known as metaphylaxis. Metaphylactic treatment of entire groups of cattle with a high risk of BRD can be highly efficacious in preventing life-threatening disease; specifically, parenteral administration of therapeutic doses of tulathromycin has been shown to be highly effective when used as metaphylaxis for preventing illness and death related to BRD (Wellman and O'Connor, 2007; Murray et al., 2016; O'Connor et al., 2016; Abell et al., 2017). However, treatment of animals with AMDs, especially mass treatment regimens, are subject to increasing concern and scrutiny because of the potential for public health impacts related to antimicrobial resistance in bacteria that may be transferred to consumers through the food chain or environmental routes.

In the most recent national survey data available, 45.3% of feedlots reporting metaphylaxis use reported using tulathromycin to prevent BRD when cattle arrived at the feedlot (USDA, 2013). Its use has been demonstrated to be highly effective in reducing BRD morbidity in feedlot cattle with only minor adverse side-effects (Modric et al., 2011; Abell et al., 2017), but cardiotoxicity has been reported in several species such as mice and rabbits (Er et al., 2011; Abdel-Daim et al., 2018). Tulathromycin is a macrolide, a class of antimicrobials considered critically important for human medicine (World Health Organization, 2011) and despite increasing scrutiny of antimicrobial use practices in livestock production, little work has been performed to study the effect of tulathromycin metaphylaxis on antimicrobial resistance (AMR) in cattle. Past research has frequently focused on phenotypic resistance to a limited number of AMDs in one, or at most a few, bacterial species using traditional culture methods (Godinho, 2008; Zaheer et al., 2013; Timsit et al., 2017). However, the response to antimicrobial use varies among bacteria and because resistance genes can be transmitted amongst a wide variety of bacteria; results found in one bacterial species cannot be extrapolated to the community level (Portis et al., 2012; Alexander et al., 2013). Such AMD exposures have potential to affect the entire gut ecology, and as such, a broader perspective is needed in investigating potential effects of metaphylactic treatment on microbial communities.

High-throughput sequencing techniques now enable a culture-independent metagenomic approach that can be used to study the resistome and microbiome, allowing access to the complete repertoire of resistance genes and bacteria within a given sample. Therefore, this study was conducted to investigate the impact of metaphylactic treatment of cattle with tulathromycin on the fecal resistome and microbiome of commercial feedlot cattle in the early feeding period using shotgun metagenomics.

## Materials and methods

### Overview of study design and population

Two groups of cattle were identified for enrollment in the study before their arrival at a commercial cattle feedlot in Texas. Cattle were purchased from a single backgrounding facility and were delivered in two groups of 193 and 186 steers (300–400 kg body weight/animal). Cattle were housed in separate pens after arrival (Day 1), and one group was randomly selected to be treated metaphylactically with parenteral tulathromycin while the other group served as an untreated control. All cattle in the treated group received a subcutaneous injection of 800 mg tulathromycin (Draxxin®; Zoetis, Florham, NJ) while cattle in the untreated group did not. This single tulathromycin treatment was expected to result in therapeutic tissue concentrations in the lung for up to 14 days (Pfizer, 2007), and this drug has a withdrawal period of 18 days in the U.S. with regard to slaughter for human consumption of tissues (FDA, 2005). Essentially all of this drug is eliminated unmetabolized from the body via biliary excretion and subsequent fecal elimination. With the exception of this treatment, both groups of cattle underwent identical arrival processing, including administration of vaccines for clostridial and respiratory diseases, avermectin anthelmintic, and application of growth-promoting hormone implants (Table 1). After initial processing and placement into pens, cattle were fed the same corn-based diet throughout 11-day study period which contained tylosin (also a macrolide class of antimicrobial) to prevent liver abscesses at an FDA approved target intake of 90 mg per head per day and ionophore feed additives (monensin) conforming to nutritional recommendations of the National Research Council (National Research Council, 2000). Cattle were provided *ad libitum* access to water and their health and welfare were monitored daily by trained feedlot personnel under the supervision of consulting veterinarians.

**Table 1:** Products administered to study cattle at the time of arrival-processing (Day 1).

**Product type**	**Commercial name**	**Manufacturer**	**Volume per animal**	**Additional information**
Antimicrobial^*^	Draxxin	Zoetis	8 cc	Macrolide antimicrobial for treatment of cattle at high risk for bovine respiratory disease (BRD).
Anthelmintic	Noromectin	Norbrooks labs	7 cc	Ivermectin parasiticide for the treatment and control of internal and external parasites of cattle.
Anthelmintic	Safeguard	Merck animal health	18 cc	For use in beef cattle for the removal and control of lung, stomach and intestine worms.
Vaccine	BoviAnthelmintic-Shield GOLD	Zoetis	2 cc	Protects cattle from infectious bovine rhinotracheitis (IBR) and bovine viral diarrhea (BVD).
Vaccine	Vision® 7	Merck animal health	2 cc	For use in healthy cattle as an aid in the preventing disease caused by *Clostridium* spp.
Steroid implant	Revalor-XS	Merck animal health	Implant	Trenbolone acetate and estradiol. It improves rate of gain and feed efficiency.

* *Only the treated group received the antimicrobial treatment*.

Fecal samples were collected from cattle per rectum at arrival (Day 1), and 11 days later (Day 11). After transport to the laboratory, fecal samples were processed to isolate total metagenomic DNA, upon which shotgun metagenomic sequencing was performed. During the 11-day study period, no cattle were identified as being ill, and therefore none received additional therapeutic AMD treatments.

### Sample collection

A total of 379 fecal samples (≥25 g/sample) were obtained per rectum from each steer at arrival processing, before tulathromycin injection for the treated group, using individually packaged sterile gloves. Each fecal sample was placed into a sterile Whirl-Pak bag (Nasco). Fecal samples were then placed into coolers with ice packs and transported to the laboratory within 8 h of sample collection for storage at −80°C. As part of another study evaluating methods for *Salmonella enterica* identification, all samples were processed prior to freezer storage with aerobic culture and lateral-flow immunoassay strips. Three cattle were identified as culture-positive for *S. enterica*; these 3 animals from the treated group and an additional 31 randomly selected animals were chosen for sampling at the second sampling time. On Day 11, these 34 cattle (17 per group) were again palpated per rectum with sterile gloves to collect feces. Four animals had minimal feces in the rectum at this time (2 per group); therefore, fecal samples were collected from 30 cattle (15 per group) and transported on ice to the laboratory for frozen storage. Thus, a total of 60 fecal samples collected at the two time points (Day 1 and Day 11) were selected for further genomic investigation and were processed for shotgun metagenomic sequencing.

### DNA extraction

The 60 fecal samples were thawed at room temperature and total DNA was isolated. To remove excess plant debris and decrease inhibitors in fecal DNA samples, 10 g from each sample were mixed with 30 mL of buffered peptone water (BPW), vigorously shaken, and allowed to sediment for 10 min. Supernatant was transferred to sterile 50 ml conical tubes and centrifuged at 4,300 × g for 10 min at 4°C. Resulting pellets were rinsed with 5 mL of molecular-grade 1X phosphate buffered saline (PBS) and centrifuged again (4,300 × g, for 10 min, at 4°C). After removal of supernatant, total DNA was isolated from the pellet using the PowerMax Soil DNA Isolation Kit (MO BIO Laboratories) following the manufacturer's protocol. DNA concentration and quality were evaluated using a NanoDrop™ spectrophotometer (Thermo Fisher Scientific, Inc.). Samples with 260:280 nm ratios >1.3 and DNA concentrations >20 ng/μl were sent for sequencing; samples that did not meet the concentration threshold were concentrated by ethanol precipitation before sequencing.

### DNA library preparation and sequencing

Purified DNA (100 μl aliquots) from all 60 samples were delivered to the Genomics and Microarray Core at University of Colorado Denver (Aurora, CO) for library preparation and sequencing. Genomic libraries were prepared using the TruSeq DNA Library Preparation Kit (Illumina, Inc.) and next-generation sequencing was completed on the HiSeq 2000 (Illumina, Inc.) with 5 samples per lane, V4 chemistry, and paired-end reads of 125 bp in length.

### Processing of metagenomic sequence data

De-multiplexed sequence reads were analyzed using the AmrPlusPlus bioinformatic pipeline (Lakin et al., 2017). Starting with read trimming and quality filtering using Trimmomatic (Bolger et al., 2014), AmrPlusPlus then identifies host DNA with alignment to the *Bos taurus* genome (Merchant et al., 2014) using the Burrows-Wheeler-Aligner (BWA) software (Li, 2013) and removes those reads with SamTools (Li et al., 2009) to create non-host reads for subsequent characterization of the resistome and microbiome.

### Analysis of sequencing quality

The FastQC software (Andrews, 2010) was used to assess sample read quality. Summary statistics regarding the number of raw, trimmed, and non-host reads for each sample were compared using generalized linear models with the “glm” function and the R platform (R. Core Team, 2013) to assess systematic bias across the following sequencing metadata: sequencing run, batch, and lane. For study design metadata, primary comparisons of interest were between treated vs. untreated cattle, and between sampling time points (Day 1 vs. Day 11). To test for potential DNA contamination, sample reads were aligned to the human genome using BWA and the number of successfully aligned reads in each sample were compared between groups using the “wilcox.test” function. Similarly, differences in sequencing results between sample groups were tested with the Wilcoxon signed-rank test when comparing paired values from the same animal (Day 1–Day 11) and the Wilcoxon rank-sum test was employed when comparing animals at either time point.

### Resistome: identification of resistance genes in metagenomic sequence data

In order to identify reads matching to resistance genes in the 60 samples, reads were aligned with BWA to the database MEGARes (Lakin et al., 2017), a non-redundant nucleotide database of publicly available AMR gene sequences. For descriptive and statistical analyses, only genes with >80% “gene fraction,” defined as the percent of nucleotides in each AMR reference gene that aligned to at least one read, were considered to be present in a sample (Supplementary Data Sheet 1). All gene accessions in the MEGARes database have been classified into an acyclic taxonomic hierarchy (drug class, mechanism, and group). Accessions in the MEGARes database that are known to cause resistance as a result of single nucleotide polymorphisms (SNPs) in genes otherwise not associated with resistance were evaluated by visualizing the BWA alignments with Integrative Genomics Viewer (Thorvaldsdóttir et al., 2013). Reads were confirmed to align to the resistant allele sequence with 100% peptide homology (to allow for silent nucleotide substitutions) across the middle 95% of the reference AMR gene. Genes identified in our samples and included in this post-processing verification step were: *parE, rpoB, phoP, phoQ, evgS, evgA, crp, evgA, envR, marA, cpxA, cpxR, ompF*, and *blaR*. Any alignments that failed this verification step were removed from downstream analyses, as those reads likely represented wild-type DNA sequences that do not confer resistance to antimicrobials. Additionally, critically important resistance determinants (when expressed in human disease-causing agents) were identified *a priori*: [bla(OXA), bla(SME), bla(IMI), bla(NDM), bla(GES), bla(KPC), bla(cphA), bla(TEM), bla(SHV), bla(CTX-M), bla(CMY), vga/vat, cfr]. Alignments to these genes accessions were specifically searched for in all 60 samples.

### Microbiome: identification and classification of bacterial sequences

Kraken (version 1) (Wood and Salzberg, 2014) was used to assign taxonomic labels to quality trimmed, paired non-host reads (Supplementary Data Sheet 2). To employ NCBI's RefSeq “Release 86” from January 12, 2018 (O'Leary et al., 2016), we created a custom kraken database consisting of RefSeq bacterial and archaeal genomes classified as either “reference genome” or “representative genome” and all complete viral genomes in RefSeq. Based on the recommendation of kraken's developers, all low-complexity regions were masked using DUST (Morgulis et al., 2006). Additionally, plasmid sequences were extracted from the genomic files and assigned to the “unidentified plasmid” taxa number ID “45202” to increase the specificity of taxonomic read classification and account for the horizontal transfer of plasmids in microbial communities (see full script at https://github.com/colostatemeg/meglab-kraken-custom-db).

### Statistical analysis

Statistical analyses of the resistome and microbiome were accomplished using R packages “metagenomeSeq” and “vegan” (Paulson et al., 2013; Oksanen et al., 2014). Sparsely represented resistome and microbiome features (genes and taxa, respectively), which were identified in fewer than 5% of samples, were removed from further analysis to reduce the likelihood that these features would bias abundance comparisons (Paulson et al., 2013). Two different methods were used to normalize resistome and microbiome feature counts. Resistome counts were normalized using an equation (Li et al., 2015) that allows for AMR gene abundance to be expressed as “copy of AMR gene per copy of 16S-rRNA gene” by accounting for differences in sequence length of AMR genes and bacterial load in the samples. Alignment to the full Greengenes database (DeSantis et al., 2006) using BWA with default settings in a paired-end manner was employed to identify 16S sequences in all non-host reads. Subsequently, the “AMR gene abundance” of each gene identified within a sample was calculated using the equation (Li et al., 2015; Supplementary Data Sheet 3):

$$\begin{array}{l} \text{AMR gene abundance}=\\ \overset{n}{\underset{1}{\sum }}\frac{N_{\text{AMR}-\text{likesequence}}\times L_{\text{reads}}/L_{\text{AMRreferencesequence}}}{N_{16\text{Ssequence}}\times L_{\text{reads}}/L_{16\text{Ssequence}}} \end{array}$$

with *N*_AMR−likesequence_ as the number of alignments to one specific AMR gene sequence; *L*_reads_ as the sequence length of the Illumina reads (125 nt); *L*_AMRreferencesequence_ as the sequence length of the corresponding AMR gene sequence; *N*_16Ssequence_ as the number of alignments to 16S sequences; and *L*_16Ssequence_ as the average length of the 16S sequences in the Greengenes database (mean = 1,401 nt). The resistome data were analyzed at the class and mechanism levels to avoid biased diversity measures caused by differences in the scientific criteria used for identification and publication of new resistance genes for different drug classes at the “gene” level (Hall and Schwarz, 2016). Alternatively, the numbers of reads that matched to microbial taxa were normalized to account for sequencing depth using cumulative sum scaling (CSS) (Paulson et al., 2013). The sparseness of count data called for using a default percentile of 0.5 for normalization based on published recommendations (Paulson et al., 2013). Corresponding taxonomic lineage for each taxon in the microbiome was identified and alignments were summed to these 6 Linnaean taxonomic levels: phylum, class, order, family, genus, and species. In total, there were 6 count matrices for the microbiome, but to reduce the repetitive reporting of results at all levels and because results at lower taxonomic levels are not considered very reliable (Peabody et al., 2015), statistical results for microbiome are presented at the phylum, class and order levels. In total, 8 unique normalized count matrices (i.e., 6 count matrices describing the microbiome and 2 count matrices characterizing the resistome) were analyzed and reported. Figures were created using the base plotting functions in R, the ggplot2 package (Wickham, 2009), and the Tableau software (Murray and Chabot, 2013).

### Ordination generation and testing

Normalized count matrices were Hellinger-transformed (Legendre and Gallagher, 2001) and used for ordination analysis with the metaMDS function from “vegan.” The metaMDS function employs non-metric multidimensional scaling (NMDS) on Euclidian distances with random starts to discover a stable ordination solution for plotting on two dimensions. Significance of separation between sample groups was tested using analysis of similarities (ANOSIM) (Clarke, 1993). To assess the degree of correlation between the resistome and microbiome, the “procrustes” function was used to superimpose metaMDS ordination graphs and minimize the sum of squared differences. In the same manner, the correlation between the untreated and treated group's microbiomes and resistomes were calculated at both Day 1 and Day 11. Then, the function “protest” was used to calculate a *M*^2^ statistic for each procrustes result (Supplementary Data Sheet 4).

### Richness and diversity comparisons

For all 8 count matrices, the richness (i.e., the total number of unique features in each sample) and Shannon's diversity (i.e., the number and proportion of unique features in each sample) were compared between sample groups using the “wilcox.test” function in R (Supplementary Data Sheet 4).

### Analysis of log-fold change in abundance

In order to identify specific features in count matrices with significantly different numbers of alignments between sample groups, metagenomeSeq's “fitZig” function (Paulson et al., 2013) was used to fit multivariate zero-inflated Gaussian mixture models for all 8 count matrices separately. To avoid spurious feature comparisons, only features present in abundances greater than the 15th quantile in each count matrix were considered. Statistical models consisted of fixed effects for sample group (e.g., treated vs. untreated, or Day 1 vs. Day 11) and sequencing batch number. The option “useMixedModel” and “block” was employed to account for repeated measures on cattle. Pairwise comparisons of feature abundance between sample groups were evaluated using limma's “makeContrasts” function (Ritchie et al., 2015) on the multivariate model, using alpha = 0.05 on adjusted *P-*values as the cut-off value for statistical significance. This function outputs an estimate of the log_2_-fold change in abundance between groups for each feature (i.e., class/mechanism/phylum/order/etc.) with an associated *P*-value adjusted for multiple comparisons using the Benjamini-Hochberg procedure (Benjamini and Hochberg, 1995).

### Data submission

Quality-trimmed sequencing reads for all 60 samples described in this project have been deposited to the NCBI collection of biological data (BioProject). Accession PRJNA309291 ID: 309291.

## Results

### Sequencing results

Across all 60 samples, shotgun metagenomic sequencing generated 5.89 billion reads (2.95 billion paired reads) with an average of 49.1 million paired-end reads per sample (range 13.49–80.36 M, Supplementary Data Sheet 5). The average Phred quality score of raw reads across all samples was 35.2 (range 34.54–35.82) and only 4.4% of all reads were removed due to low quality (minimum per sample = 2.48%, maximum = 8.21%). Of the remaining reads, 19.69% (55.44 M reads) were identified as bovine DNA and removed from subsequent analysis; 3 samples contained nearly 37% bovine DNA (probably because the feces were relatively scant in the rectum of these cattle at the time of sampling) and the other 57 samples ranged from 19.69 to 27.11%. Alignment of non-host reads to the human genome identified on average 991,958 reads per sample (range = 210,246–4,639,154) and suggested minimal sample contamination (2.6% of reads across all 60 samples). There was a small, statistically significant difference in Phred scores when comparing samples by time and treatment due to high quality reads in all 60 samples (mean = 35.23, range = 34.54–35.82). This difference was not considered to be biologically meaningful. Additionally, because no other metadata comparisons yielded statistically significant differences, our results suggested that there was no systematic bias in sequencing effort.

### Resistome composition

**Figure 1:**
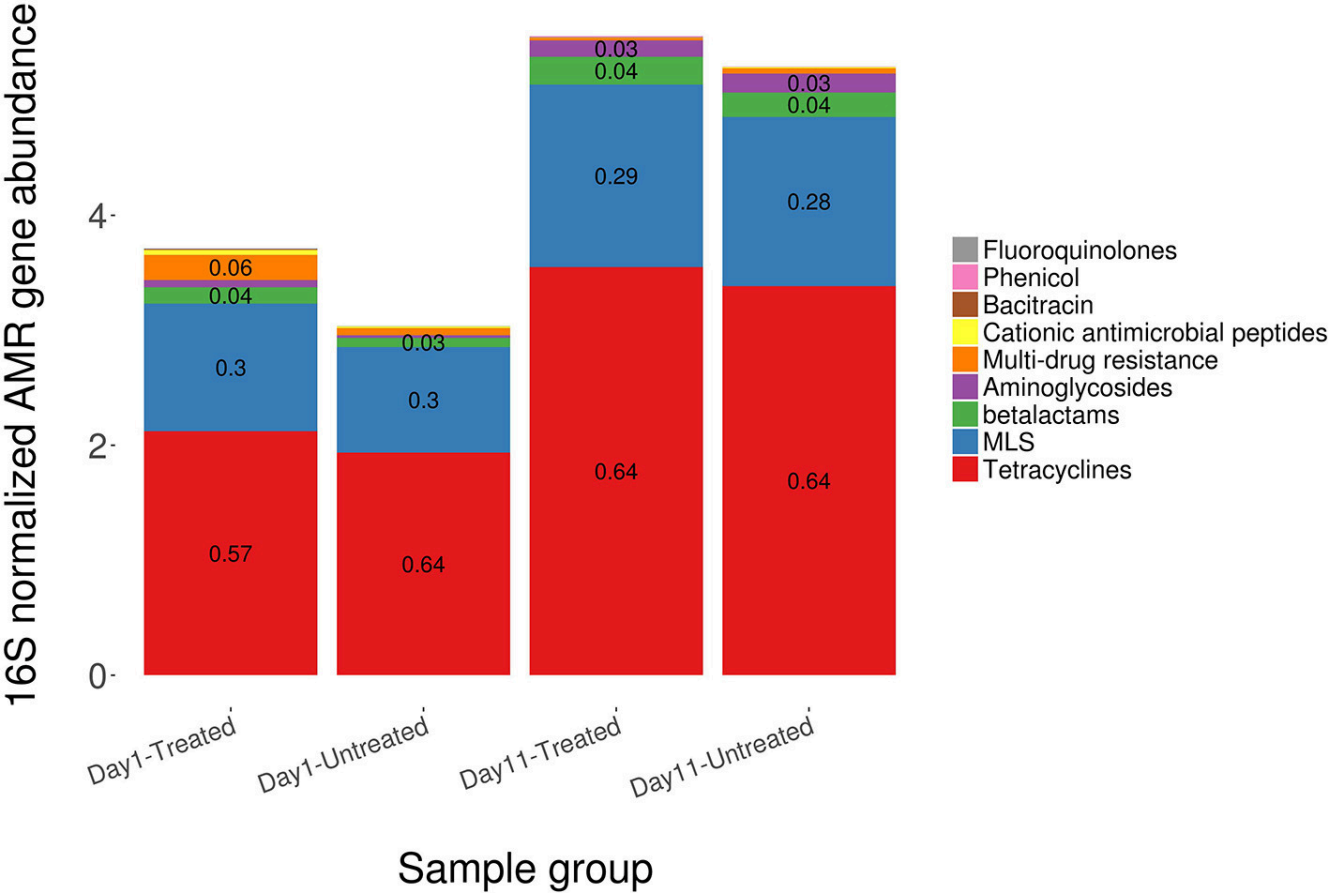
Total AMR gene abundance determined by shotgun metagenomic sequencing and normalized using 16S rRNA abundance, by drug class, among treated and untreated cattle in samples obtained at Day 1 and again at Day 11. Values are formulated from the number of reads that aligned to AMR genes and normalized to bacterial abundance characterized by alignments to 16S gene sequences from the Greengenes database.

4,054,637 reads aligned to 208 AMR gene accessions in the MEGARes reference database. Following confirmation of genes conferring resistance due to single nucleotide polymorphisms (SNPs) and removal of sparsely represented genes (i.e., those found in <3 samples), there were 134 unique gene accessions in the MEGARes database that were identified from 3,773,873 reads. In all, these represented resistance to 9 unique AMR drug classes via 24 mechanisms of resistance, though the clear majority of reads aligned to gene accessions that confer resistance to tetracycline and the macrolide-lincosamide-streptogramin (MLS) class of antibiotics (76 and 18% of aligned reads, respectively). More than 99% of the reads that aligned to tetracycline resistance gene accessions are known to confer resistance through ribosomal protection proteins, and 77% of the reads that aligned to MLS resistance gene accessions are known to confer resistance through macrolide efflux pumps. The remaining AMR features were identified in low abundance across all study samples and consisted of gene accessions associated with multi-drug resistance (e.g., non-specific multi-drug efflux pumps) and resistance to the following drug classes; phenicol, bacitracin, fluoroquinolones, cationic antimicrobial peptides, aminoglycosides, and betalactams. This pattern of fecal resistome composition was observed in both study groups and was seen in samples collected at both Day 1 and Day 11 (Figure 1). Of the *a priori* identified critically important resistance determinants, we only identified one AMR gene accession, bla(CTX-M), in a single sample from the treated group on Day 11.

**Figure 2:**
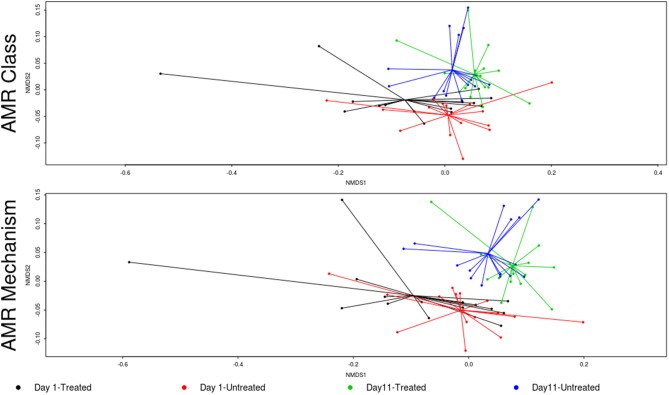
Ordination comparing resistome composition at the AMR drug class and resistance mechanism, using non-metric multidimensional scaling (NMDS), for the two study groups at Day 1 and Day 11. Separation of resistomes from treated and untreated cattle was not statistically significant at either Day 1 or Day 11 (Day 1 vs. Day 11; ANOSIM *P* > 0.05). However, the resistomes of the treated and untreated groups were significantly separated over time (Day 1 vs. Day 11; ANOSIM *P* < 0.05).

The overall resistome composition was similar between the treated and untreated groups at both Day 1 and Day 11 (Figure 2). Apart from alignments to tetracyclines and MLS gene accessions, <3% of the resistome was characterized by alignments to multi-drug, betalactam, and aminoglycoside resistance gene accessions, with alignments to remaining classes of drugs each accounting for <1% of all alignments. While we did identify a difference in AMR Shannon's diversity when comparing treated and untreated cattle at Day 1 (*P* = 0.05), there was no evidence of significant differences in the relative abundances of AMR classes or mechanisms. In contrast by Day 11, the untreated group had significantly different AMR richness at the mechanism level (*P* = 0.02) and contained significantly higher abundance for the AMR mechanism, Tetracycline inactivation enzymes, than the treated group (*P* < 0.05).

**Figure 3:**
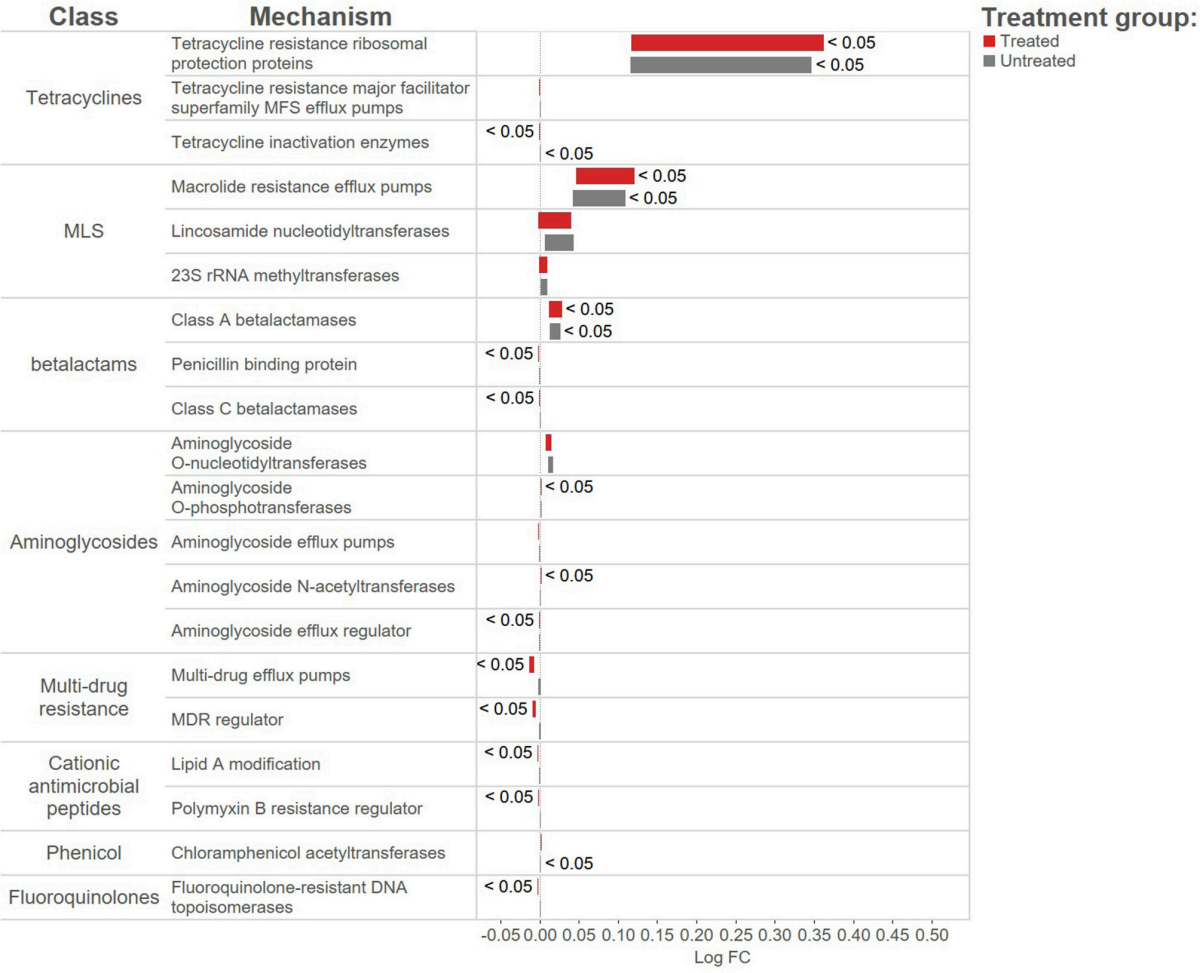
Log-fold change in abundance to AMR mechanisms for the treated (red bars) and untreated (gray bars) over time from Day 1 to day11. Bars to the right of the 0-line signify an increase in abundance, the size of the bars represent the average expression of the AMR mechanism and bars are labeled with adjusted *p*-values < 0.05.

**Figure 4:**
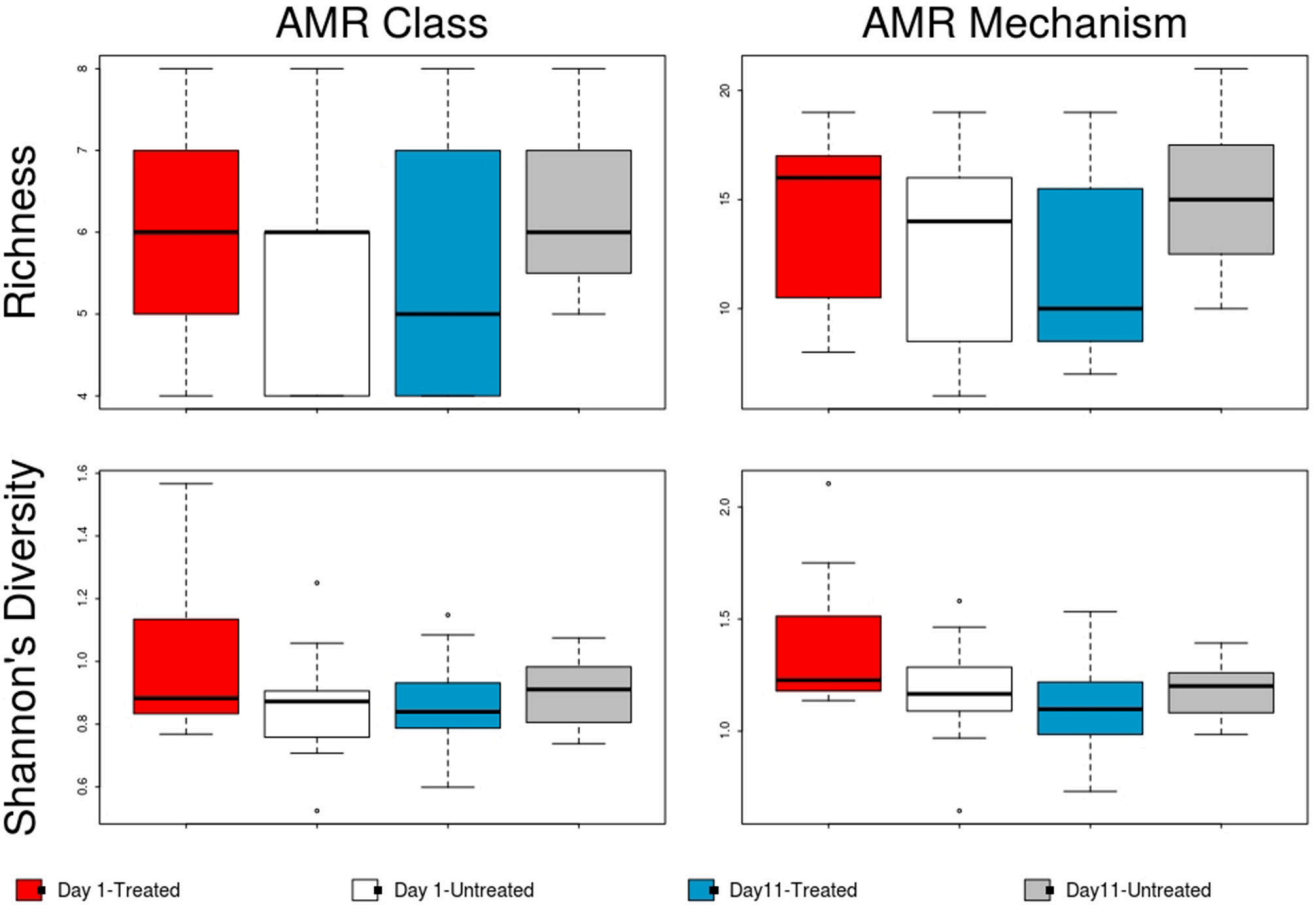
Boxplot of resistome richness and Shannon's diversity at the AMR class and mechanism levels of the two study groups at Day 1 and Day 11. The horizontal line is the median value, the middle box indicates the inter-quantile range, whiskers represent values within 1.5 IQR of the lower and upper quartiles, and individual points show outlier values.

In contrast to the lack of difference between treated and untreated groups at both time points, there was a dramatic change in the resistome of both groups between Day 1 and Day 11, such that there appeared to be a convergence toward a “common” resistome between groups. The untreated group's resistome shifted significantly at the class (ANOSIM *R* = 0.22, *P* = 0.002) and mechanism levels (ANOSIM *R* = 0.30, *P* = 0.001), as did the resistome of the treated cattle (ANOSIM *R* = 0.21, *P* = 0.001 for AMR drug class and ANOSIM *R* = 0.40, *P* = 0.001 for AMR mechanism) (Figure 2). In both study groups, total AMR abundance, defined as “copies of alignments to AMR gene accessions per copy of 16S-rRNA gene”, increased over time (Day 1–Day 11) from 3.04 to 5.29 in the untreated group and from 3.71 to 5.56 in the treated group. Consequently, the relative abundance of alignments to the two most abundant AMR classes, tetracyclines and MLS, increased between Day 1 and Day 11 for both the treated and untreated groups (*P* < 0.05). The untreated group's resistome increased in abundance in two additional AMR classes, aminoglycoside and betalactam resistance (*P* < 0.05) albeit without exposure to these drugs. Correspondingly, the untreated group's significant changes in abundance were all increases in relative abundance of alignments to 5 of 20 resistance mechanisms between Day 1 and Day 11 (*P* < 0.05). Alternatively, the treated group had 15 mechanisms with significant changes in abundance, but 10 of 15 mechanisms decreased in abundance over time (Figure 3). Three AMR mechanisms increased in relative abundance in both groups, including tetracycline resistance ribosomal protection proteins, macrolide resistance efflux pumps, and class A betalactamases. The other 2 AMR mechanisms that increased in abundance over time differed by treatment group; aminoglycoside O-phosphotransferases and aminoglycoside N-acetyltransferases in the treated group, compared to increases in alignments to tetracycline inactivation enzymes and chloramphenicol acetyltransferases in the untreated group. Shannon's diversity indices of the treated group at the mechanism level decreased significantly over time (*P* = 0.04), whereas there were no significant changes in richness or Shannon's diversity in untreated group (Figure 4). During these shifts in the resistome over time, procrustes analysis suggests that class level AMR resistome composition of treated and untreated cattle became more similar as they were significantly correlated only at Day 11 (*M*^2^ = 0.71, *P* = 0.02).

While major trends in the most abundant AMR features can be observed at the treatment group level, there was considerable variation in the presence of low abundance AMR mechanisms between animals (Supplementary Image 1). Interestingly, the number of samples with alignments to phenicol and glycopeptide AMR classes increased over time in both study groups, though differential abundance comparisons were not possible due to their low abundance and sparse representation across all 60 samples. There were no samples with phenicol resistance gene accessions at Day 1, but alignments were present in 8 of 15 cattle from each treatment group by Day 11. Similarly, no samples had alignments to glycopeptide resistance gene accessions at Day 1; however, by Day 11 glycopeptide class resistance genes were identified in 3 of 15 untreated animals. It is important to note that no glycopeptide antimicrobials had ever been used in these cattle or in this facility, as this is illegal in cattle production in the U.S.

### Microbiome composition

**Figure 5:**
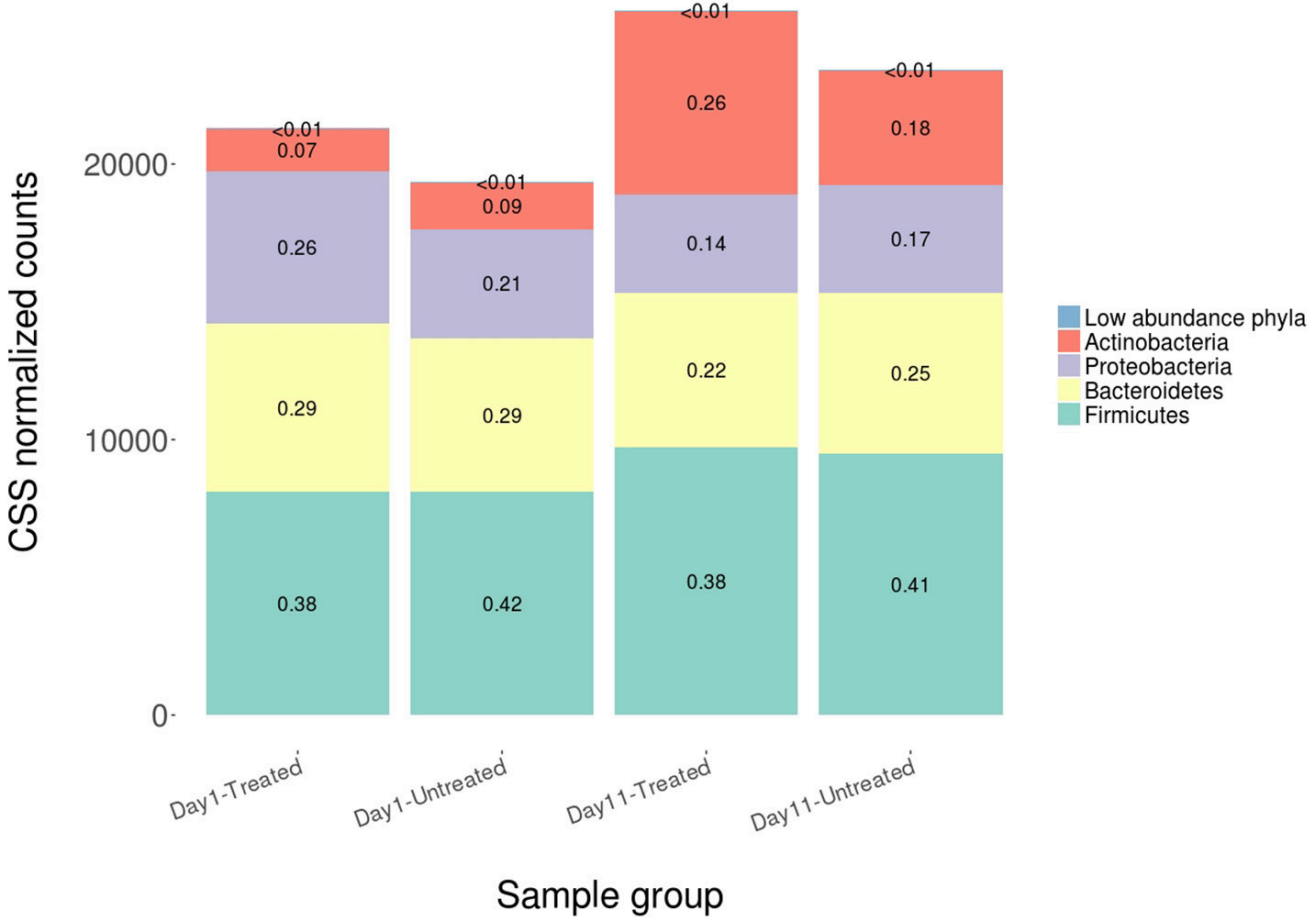
Average relative abundance of CSS normalized counts of shotgun metagenomic reads aligning to bacterial, archaeal and viral genomes at the phylum level for both study groups at Day 1 and Day 11. Phyla comprising <3% of each sample group were combined into the category “Low abundance phyla”.

On average, 96.14% of sample reads were not classified as bacteria, archaea, or viruses (range = 93.71–96.98%). Alignments to a total of 5,910 taxa were identified across the 60 samples. Sparsely represented taxa were removed prior to normalization such that a total of 5,383 unique taxa were included in subsequent analyses (comprising alignments attributed to 38 phyla, 74 classes, 170 orders, 384 families, 1,211 genera and 3,943 species). The majority of microbiome alignments were to bacteria; alignments to Firmicutes, Bacteroidetes, Proteobacteria, and Actinobacteria were most common, accounting for 99.7% of the total normalized hit counts at the phylum level (37, 24, 18, and 15%, respectively). At the class level, Clostridia (29%), Bacteroidia (21%), Gammaproteobacteria (10%), and Coriobacteriia (7%) were the predominant classes to which alignments were classified, representing more than two thirds of normalized counts. Clostridiales (32%), Bacteroidales (21%), and Enterobacteriales (6%) were the most abundant taxa at the order level (Figure 5).

**Figure 6:**
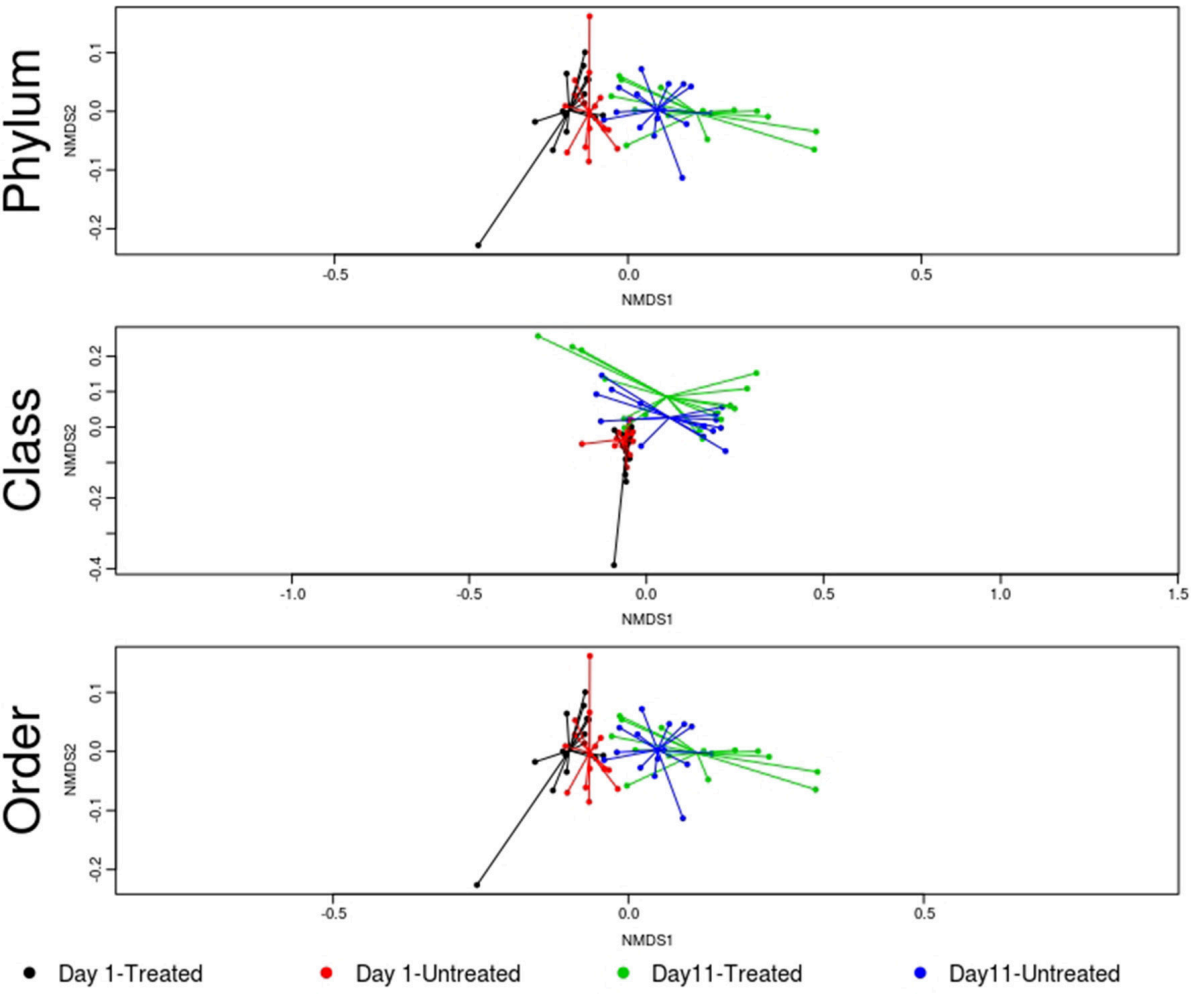
Ordination comparing microbiome composition at the phylum, class, and order levels, using non-metric multidimensional scaling (NMDS), for treated and untreated groups of cattle at Day 1 and Day 11. Separation of microbiomes from treated and untreated cattle was not statistically significant at either Day 1 or Day 11 (treated vs. untreated; ANOSIM *P* > 0.05). However, microbiomes for the study groups differed significantly over time (Day 1 vs. Day 11; ANOSIM *P* < 0.05).

**Figure 7:**
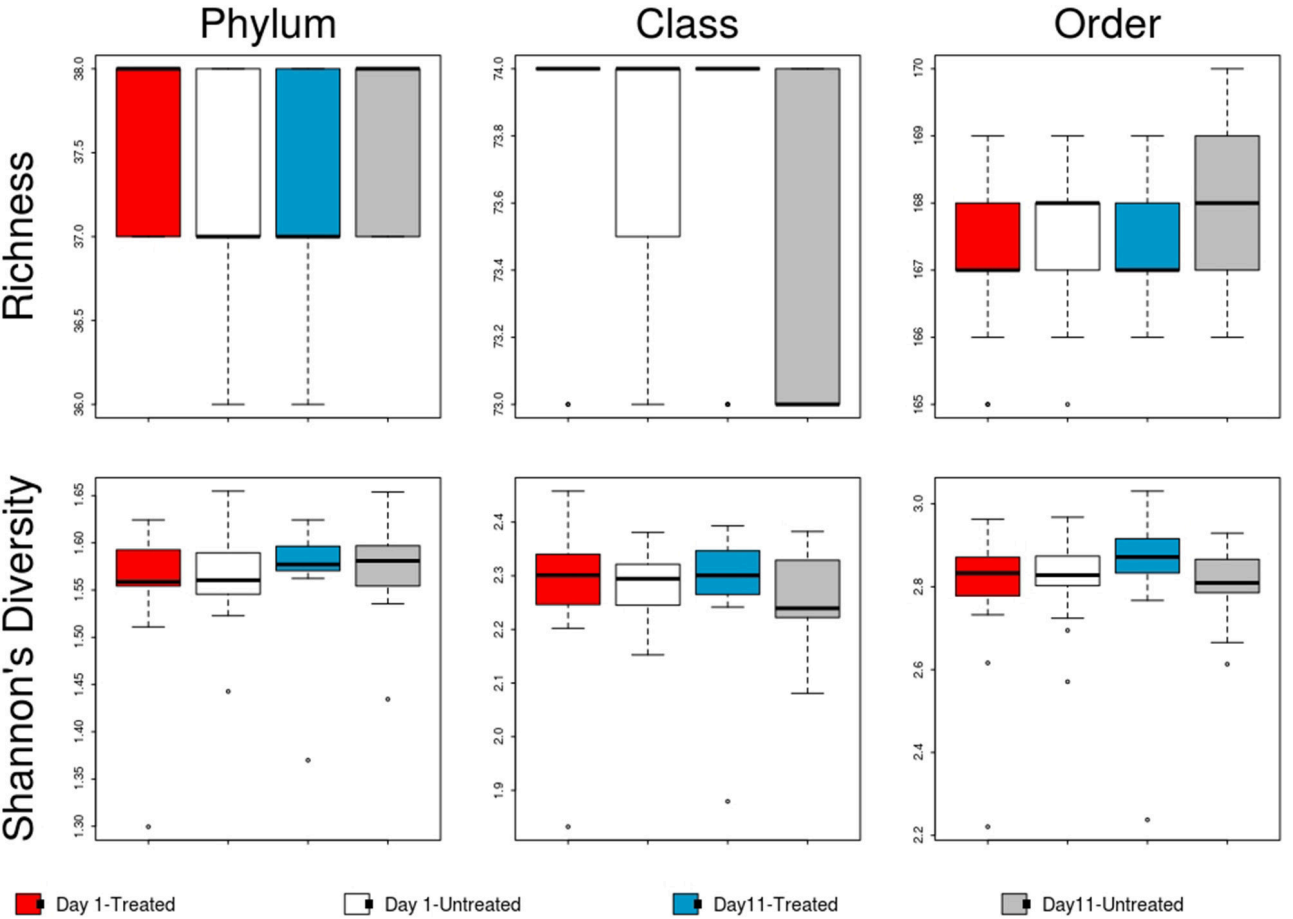
Boxplot of microbiome richness and Shannon's diversity at the phylum, class and order levels of the two study groups at Day 1 and Day 11. The horizontal line is the median value, the middle box indicates the inter-quantile range, whiskers represent values within 1.5 IQR of the lower and upper quartiles, and individual points show outlier values.

No significant differences in the overall microbiome were observed between treated and untreated groups at Day 1 (ANOSIM *P* > 0.05), and taxa were not differentially abundant at the phyla, class, or order level after adjusting for multiple comparisons. Similarly, at Day 11, ordination comparisons showed no distinct separation of microbial communities between the treated and untreated groups (Figure 6), and relative abundance of microbiome features did not differ at the phyla, class, or order levels. Moreover, richness and Shannon's diversity did not differ significantly between groups at either Day 1 or Day 11 (Figure 7). Unlike the resistome, procrustes analysis did not identify significant correlations between the groups' microbiomes at either time point.

Despite evidence suggesting that both groups had similar fecal microbiomes at Day 1 and Day 11, the composition shifted significantly over time in the feedlot at all microbiome levels for both the untreated group (phylum level: ANOSIM *R* = 0.51, *P* = 0.001) and the treated group (phylum level: ANOSIM *R* = 0.50, *P* = 0.001). The major shift that occurred in the composition of both study groups' microbiomes between sampling dates was characterized by an increase in the proportion of Actinobacteria and Firmicutes, which together accounted for 58% of the untreated and 64% of the treated group's resistome at Day 11 compared to 51 and 45% at Day 1, respectively (Figure 5). In the treated group, 17 of 38 phyla show significant changes in abundance over time, although there were only shifts in 7 of 38 phyla in the untreated group. Both groups' microbiome significantly increased in relative abundance of Firmicutes and Actinobacteria phyla, combined with a decrease in relative abundance of Gemmatimonadetes, Euryarchaeota, Candidatus Saccharibacteria, and Candidatus Planctomycetes (*P* < 0.05). Of the remaining phyla with significant changes in the treated cattle, 4 of 10 taxa increased in relative abundance, while the other 6 phyla decreased in abundance (*P* < 0.05). Notwithstanding the major changes in microbiome composition, neither richness nor Shannon's diversity measures changed over time in either group.

### Relationships between the fecal resistome and microbiome

Procrustes analyses suggests no statistically significant correlations were present between the resistome and microbiome within treatment groups at either time point (*P*> 0.05).

## Discussion

Results of this study suggest that parenteral metaphylactic treatment of cattle with tulathromycin had minimal, if any, detectable short-term impact on the fecal resistome and microbiome of commercially raised feedlot cattle when evaluated using shotgun metagenomic sequencing. This is important because of critical concerns about public health in relation to AMD use in food-producing animals and also because this is an important drug for treatment and control of life-threatening respiratory disease in feedlot cattle. This study was conducted in a commercial feedlot operation to improve the practical relevance of our findings, but this also introduces important limitations. USDA data suggests that over 70% of feedlot cattle in the U.S. receive low doses of tylosin, a macrolide drug, in-feed for prevention of liver abscesses (USDA, 2013). While tylosin exposure of all study cattle may have confounded our ability to independently investigate the effects of tulathromycin (a different macrolide drug), this study aims to characterize the effect of additional metaphylactic treatment in the context of commercial feedlot cattle production systems. Likewise, other studies have described that parenteral treatment with a tetracycline drug (oxytetracycline) can cause discernible changes in AMR even when cattle are also exposed to another in-feed tetracycline AMD (chlortetracycline) (O'Connor et al., 2002; Holman et al., 2018). Comparing fecal samples collected at Day 1 to those collected on Day 11 uncovered several notable changes in the resistome and microbiome, suggesting that the transition from backgrounding operations to concentrated feeding in a commercial feedlot is a critical time for influencing the microbial community of beef cattle. The ancient phenomena of AMR is not likely to be eliminated from microbial communities in natural environments (D'Costa et al., 2011), so techniques used to manage food animal populations (e.g., AMD use, diet changes, prebiotics, probiotics) need be evaluated as a way to support animal health and productivity while reducing AMR prevalence and transmission (Gaggìa et al., 2010; McCann et al., 2014). This study provides an ecological perspective suggesting metaphylactic tulathromycin treatment may be employed without incurring drastic changes to the resistome and microbiome of typical feedlot cattle.

Between treated and untreated groups, shifting abundance from Day 1 to Day 11 in resistome and microbiome features differed by treatment, but ultimately maintained a “common” composition and total AMR abundance comprised principally of relatively few, highly-abundant taxa. In particular, procrustes analysis for the correlation between the groups' resistome was only significant at Day 11. Further, the resistome and microbiome of treated and untreated groups were largely similar on Day 11, suggesting that other selective pressures besides tulathromycin metaphylaxis (e.g., common environmental exposures, exposure of all study cattle to in-feed tylosin) are potentially stronger influences on changes to the resistome and microbiome in cattle that have been newly introduced to the feedlot environment. Limited sample size of 15 animals per treatment group could limit statistical power to detect differences in the resistome and microbiome caused by tulathromycin exposure, but a search of the relevant literature indicated a lack of power calculation tools for shotgun metagenomic sequencing experiments. The difference in Shannon's diversity observed between treated and untreated cattle could have occurred because individual cattle randomization into the two pens was not logistically feasible due to constraints imposed by the feedlot operator. Specifically, to address logistical complexities in cattle production, the cattle in this study were shipped in two separate container trucks from the backgrounding facility, and these separate groups automatically became the treated and untreated groups upon arrival in the feedlot, as they were housed in separate pens due to arrival processing considerations. Nevertheless, this study contributes an ecological perspective into the microbial communities of individual feedlot cattle and emphasizes the utility of studying the bacterial community in beef feedlot operations to better characterize AMR dynamics.

This study verifies past reports that tetracycline and MLS resistance is commonly identified in cattle environments (Ghosh and LaPara, 2007; Chen et al., 2008; Platt et al., 2008; Kyselková et al., 2015). Consistent with our group's previous research, resistome composition was largely dominated by the abundance of sequence alignments to two mechanisms of resistance, representing tetracycline (ribosomal protection proteins) and MLS (macrolide efflux pump) classes of resistance which accounted for >60% and >28% of resistance in the treated and untreated study groups (Noyes et al., 2016a,b). It is notable that there were no other parenteral antimicrobial drug treatments because of illness in the study cattle prior to Day 11, including a lack of exposure to drugs commonly used to treat illness in feedlot cattle such as tetracyclines, betalactams, and fluoroquinolones. It is possible that this influenced the decrease in alignments to AMR gene accessions in samples from both groups that encode for resistance to drugs not used in the study, such as bacitracin and fluoroquinolone. Interestingly, glycopeptide drug use is prohibited in beef cattle in the U.S., and while resistance was not identified at Day 1, three animals in the untreated group contained alignments to glycopeptide gene accessions. Similarly, chloramphenicol resistance was not identified at Day 1 and despite study cattle not being exposed to chloramphenicol drugs, at Day 11 both groups of cattle had 8 of 15 animals with alignments to chloramphenicol resistance gene accessions.

For the microbiome, time in the feedlot from Day 1 to Day 11 was associated with significant shifts in the microbial population in both groups, though ultimately maintained similar composition that was dominated by Firmicutes, Bacteroidetes, Proteobacteria, and Actinobacteria. Temporal changes in the microbiome of cattle acclimatizing to feedlot rearing have reported dramatic changes in the nasopharyngeal microbiota of beef cattle after arrival at a feedlot (Holman et al., 2015; Timsit et al., 2016). These shifts in the fecal microbiome might be expected given the changes cattle are experiencing after arrival to the feedlot. In the microbiome of both groups, for example, we detected an increase of typical carbohydrate-digesting bacteria such as Lactobacillales, along with an increase of organisms with diverse metabolic functions within the phyla Firmicutes from Day 1 to Day 11 (Fernando et al., 2010; Petri et al., 2013; Yang et al., 2016). Notably, the exposure to tulathromycin might have caused the decrease in relative abundance to the Proteobacteria and Verrucomicrobia phyla in the treated group. Both phyla consist of gram negative bacteria not typically considered macrolide targets, but their decrease in relative abundance is associated with concurrent increases in Firmicutes as reported with exposure a different macrolide drug, azithromycin (Parker et al., 2017). This corroborates previously published data asserting that microbiome similarity between cattle is strongly driven by exposure to comparable management practices and/or the same geographic region (Shanks et al., 2011).

Though we were not able to obtain information about the management of study cattle prior to arrival at the feedlot (i.e., source of cattle, diet, antimicrobial use, etc.), the lack of major differences in the resistome between groups at Day 1 might be attributed to rearing in the same backgrounding facility under similar husbandry practices immediately prior to being shipped to the feedlot. It is important to note that the lack of difference between treated and untreated groups either at Day 1 or Day 11 could also be explained by the high abundance of sequences (>90% relative abundance) coding for resistance to tetracyclines and MLS making a “core” resistome which could potentially mask important differences in less abundant resistance genes (Chambers et al., 2015). The pharmacokinetics of tulathromycin tissue concentration have been previously described (Evans, 2005), so the choice of 11 days between sampling points ensured that tulathromycin was still in therapeutic concentrations, but its influence on the fecal resistome and microbiome is undefined and future studies should consider time series sampling to capture temporo-dynamic changes in AMR ecology. Future research is needed to estimate the risk of different resistome compositions compared to our understanding from AMR patterns found in certain pathogens through traditional culture-based approaches. Additionally, while sequencing processes and bioinformatic analyses techniques continue to improve, we need broad collaboration from the scientific community to standardize AMR gene nomenclature and bioinformatic analysis so that results can be comparable across studies (Hall and Schwarz, 2016; Quince et al., 2017).

## Ethics statement

This study was evaluated by Colorado State University's Research Integrity and Compliance Review Office and deemed exempt from IACUC oversight (#2015-002).

## Author contributions

KB and PM designed this study, obtained funding, secured partnerships with industry partners where the study was conducted, and provided oversight for all other aspects of the study. ED and PR administered experimental treatment and collected samples. ED, PR, BB, XY, MW, LL, and RM performed sample processing and laboratory testing. ED, PR, KB, NN, CB, and JR oversaw and performed bioinformatic analysis. ED, PR, and NN contributed equally to analyzing the data and drafting the manuscript. All authors read, edited, and approved the final manuscript.

### Conflict of interest statement

The authors declare that the research was conducted in the absence of any commercial or financial relationships that could be construed as a potential conflict of interest.

## Supplementary material

Supplementary Data Sheet 1 AMR gene accession raw count of alignments.

Supplementary Data Sheet 2 Microbiome raw count of alignments.

Supplementary Data Sheet 3 AMR counts normalized using 16S counts.

Supplementary Data Sheet 4 ANOSIM, Wilcox, and Procrustes results.

Supplementary Data Sheet 5 Sequencing results and metadata for all 60 samples.

Supplementary Image 1 Heatmap of log-normalized abundances for each AMR mechanism indicated by increasing shade of blue and the number of samples containing the mechanism in each sample group (*n* = 15 per group).
