# Supplementary figures and images for: Investigating Effects of Tulathromycin Metaphylaxis on the Fecal Resistome and Microbiome of Commercial Feedlot Cattle Early in the Feeding Period
Source: Front Microbiol. 2018 Jul 30;9:1715. doi: 10.3389/fmicb.2018.01715 (PMC6077226; doi:10.3389/fmicb.2018.01715)

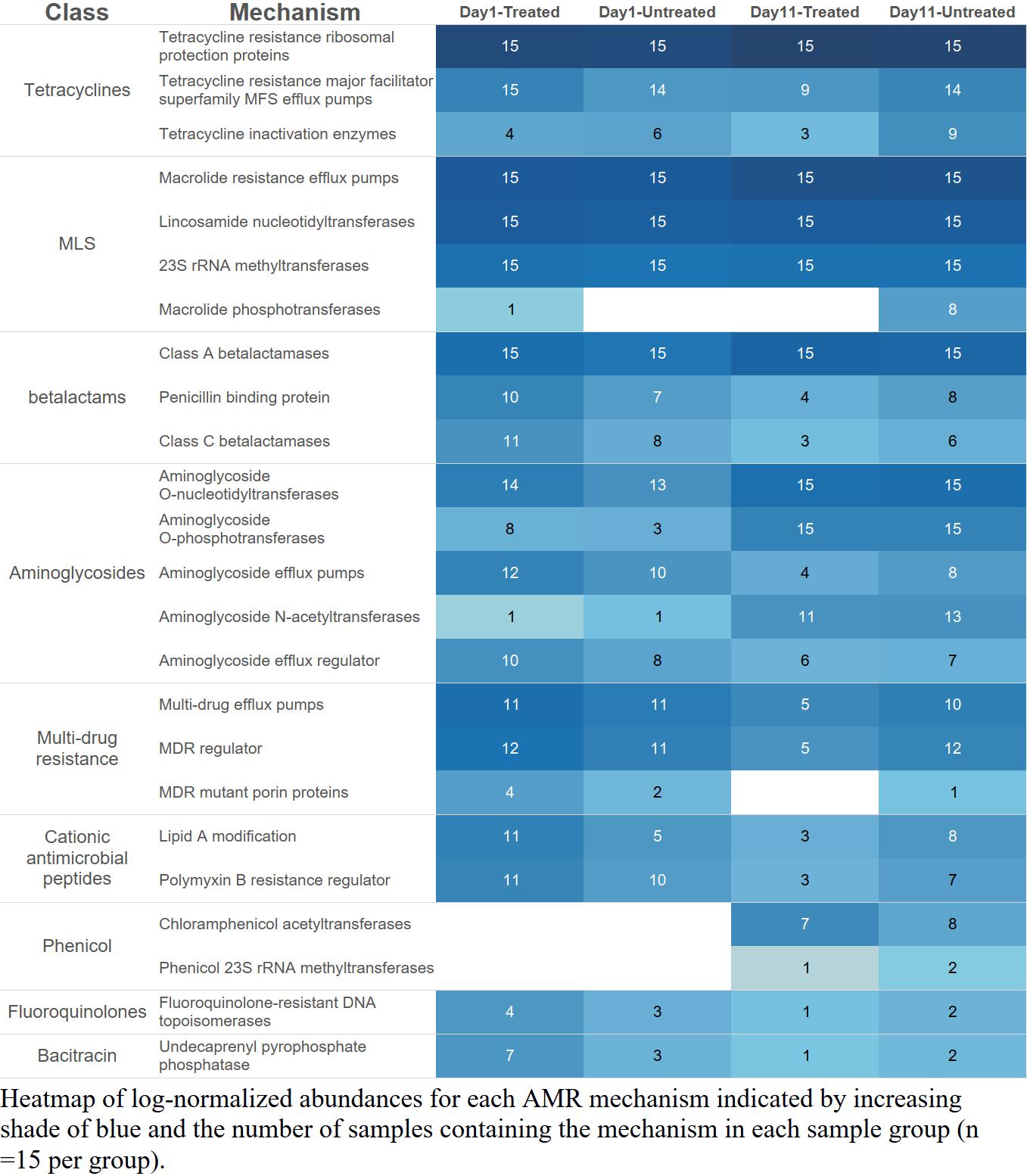

Supplement: Supplementary Image 1 — Heatmap of log-normalized abundances for each AMR mechanism indicated by increasing shade of blue and the number of samples containing the mechanism in each sample group (n = 15 per group). [file Image_1.JPEG]
